# Psychological Quarterly Retrospect

**Published:** 1860-01-01

**Authors:** 


### *Psychological Quarterly Retrospect.*

It is recorded of Heimdall, the warder of the Scandinavian gods, that the subtlety of his ear was so great that he could hear the grass growing in the meadows, and the wool on the backs of the sheep. There is a segment of society who, lacking this acuteness of sense and perception, deny or doubt the progressive intellectual and moral growth of the nation. They cannot trace this growth from day to day; nay, so far as their observation extends, the nation appears to be, if anything, deteriorating. This, it is said or implied, may be gathered from the experience of a single life-time. Whom have we now comparable with the mental giants who lived in the days of our childhood? When did society ever exhibit such signs of rottenness at the core as it now does? The policeman culls his choicest criminals freely from the most favoured classes—nay, even from the elect among them; and Sir Cresswell Cresswell enacting, as it were, the part of Asmodeus, unveils before us almost daily a picture of our domestic morality—a picture of the inner workings of the moral life of society, which is abnormal in the highest degree. These objections are about as pertinent as if a farmer were to deny the progressive development of his crops, or the thickening of the fleeces on his sheep, because he could not mark distinctly from hour to hour, or day to day, the growth of each individual blade of grass or corn, or of every lock of wool; or if, as he advanced in experience and knowledge, and became more apt in distinguishing defects or diseases in his fields and herds, he were to conclude, that therefore these defects and diseases were on the increase; or if, finally, he were to assert that the farming of his childhood and of his forefathers was more rational than the present system. The farmer knows that the splendid beeves which traverse his meadows are not the result of one generation's development; that the crop does not come to maturity coincidently with the tilling of the ground and the scattering of the seed; but that its growth and ripening are matters of time and season. And so of the moral and intellectual growth of nations. This is not a question of a life-time, but of generations. He who thinks that he may mark off the effects of mental culture on a race year by year, is in a state of ignorance as deep, but, truly, not so happy as that of the child whose imagination being warmed by the wondrous adventures of the glorious Jack surnamed of the Beanstalk, throws a bean out of the window when it goes to bed at night, expecting when it rises in the morning to see the plant grown even up to the heavens.

This is, indeed, the stamp of thinker who indulges in incredulous sneers at the various movements tending towards the mental and physical culture of the people, represented by the Social Science Association. And yet the Association itself, both in its history and objects, might have been expected to check any such immature thinking. The society is only three years old, and it constitutes the first practical attempt to give coherency of aim to the many elements which are requisite to raise man's moral and intellectual standard. It is a means to an end, and its formation was only practicable when its necessity became felt. It took many years' labour before that necessity was appreciated among even the educated classes; hence when the Association was formed, it was the sign of an important advance made. The formation, moreover, and the objects of the Association implied a want of unison, and a consequent deficiency in power, in the efforts it represented, and to facilitate and perfect the operation of which it was constituted. The Association, in short, marks the termination of the nonage of the great efforts which are being made for the physical and intellectual culture of the people, and of itself should have been a sufficient caution against hasty conclusions to those writers who have neglected history, to judge of the progress of a nation by the gauge of their individual experience.

If we would rightly estimate the moral and intellectual growth or decadence of a nation, we must turn to history, and in that of our own country we may read a story of persistent growth throughout many centuries in all that is noblest in humanity—a growth which we have just reason to believe is more vigorous now than at any previous period of the nation's existence.

At the last meeting of the Social Science Association, Sir J. K. Shuttleworth read a paper on Social Economy, in which he recounted the history of Civilization in England. By this history he endeavoured to show that the progress of nations, like that of nature, is characterized by successive eras of development. "Each era," he said, "is marked by the operation of some new force on our domestic and social habits, internal organization, civil or religious policy—all tending to produce that form of civilization which we now enjoy." He supported this thesis to demonstration by a masterly and invaluable epitome of the history of social economy in the kingdom. It is impossible to give any abstract of Sir J. K. Shuttleworth's paper, but we quote a protion of his account of the last stage of social growth which the nation has manifested :—

"Two strong tendencies have accompanied the growth of the population from 6,000,000 in 1700 in England and Wales to upwards of 20,000,000 at the present time. The first has been the growth of great towns (some of which have increased their population six or eightfold), the aggregation of the people on the coal-fields, by migration from the agricultural and moorland districts and from Ireland, as well as by the extraordinary stimulus given to natural increase by higher wages, early employment, and early marriages. The second has been that hitherto the sudden checks in commercial prosperity caused by wars, by defective harvests, by the errors of our commercial legislation, by the very eagerness of enterprise, blindly glutting foreign markets, have all been met by the silent influence of colonization, which in dangerous crises have enriched our colonies and the United States with the transiently surplus labour of the United Kingdom. There is scarcely a more touching incident in our national history than the fact that the Irish emigrants to North America have, since the failure of the potato crop and the famine of 1846, sent 9,000,000*l*. sterling to their relations in Ireland to enable them to follow. The migration, immigration, and vast increase in the numbers of the people—their gathering from comparative isolation in rural districts to close aggregation on the coal-fields under the stimulus of higher wages—began at a period when the schooling of the common people had been uncared for. Raikes commenced his Sunday school in Gloucester in the latter end of the last century. The people, during fifty years of the last, and twenty of the present century, were chiefly educated in the workshop. To the training of skilled work was superadded, with the growth of the factory system, that organization for the division of labour requiring a subordination of ranks in every mill according to capacity and skill. Hence grew habits of punctuality, order, the economy of time, the strict care of property, and exact obedience to discipline. Every factory has resembled in those respects a regiment; but it has included more gradations of rank, and a still more complicated system of submission to authority. The hand, the eye, the mind, have been trained to the most rapid manipulations; the character of the people has been gradually moulded by the great institutions of industry. This in part explains the comparative order with which such vast social changes have occurred among an otherwise rude and uninstructed people. The Legislature was ignorant of the true principles of finance and of commercial legislation to such an extent that, but a century ago, it prohibited by sumptuary laws the wearing of printed calicoes; and, with a limited area of culture, until a very recent period, deprived our vast mechanical force of the opportunity to purchase cheap bread. We should not, then, wonder at those outbursts of the fury of a population whom we had neglected to instruct, and who met each severe though transient embarrassment in the labour market by the "Luddite" riots of Yorkshire, and the successive efforts to destroy the spinning-jenny, the carding-machine, and the power-loom in Lancashire. Our workpeople, taught rather by the press, by the influence of public opinion, and by personal experience, than as yet acquainted with any abstract principles, are ready now to protect the machines which they formerly destroyed. In like manner, the attempt to regulate wages by associations of workmen has, under similar influences, undergone successive forms of amelioration. Secret associations, bound by terrible oaths, and issuing orders of assassination, are apparently not extinct, if the recently reported attempts in Sheffield are to be received as evidence of the existence of these ruthless gangs. But the recent strikes in Preston and other parts of Lancashire have passed off without tumult and without the picketing of mills. There remain forms of interference with personal freedom of workmen almost as effectual. Moral intimidation has succeeded to violence, the isolation of the 'knobstick' or 'black sheep' who will not join the union, is substituted for personal assaults; the Trades Unions attempt to dominate in their own class by refusing to work with 'knobsticks' or 'black sheep,' and thus to subordinate the whole body of the workmen in any trade to the orders of the directing committee. If the representative system of such associations were perfect; if the committee were always disinterested and pure; and if they had a more comprehensive knowledge of general principles than they have ever shown, it is clear that this social control of individual action would constitute at best that tyranny of the majority which De Tocqueville has conclusively shown to be the fatal crime against public and private liberty of any purely democratic constitution. But I have not only an unwavering confidence that these are transient forms of evil, they are even signs of an advancing civilization. They are irregular and disturbing movements of a great social force, slowly, but with the certainty which marks the great operations of nature, adjusting the relations of labour and capital, so as to be consistent with that partnership between the free and intelligent workman and his employer for which step by step our whole history has been a preparation. Nevertheless, great evils remain to be corrected. The first effect of a rise of wages among a rude people has commonly been the increase of the coarser forms of sensual enjoyment. Thus, Mr. Porter, some years ago, estimated the amount of beer, spirits, and tobacco consumed by working men in the United Kingdom at 53,411,615*l.*; the spirits amounting to 20,810,208*l.*, the beer and porter to 25,383,165*l.*, and the tobacco and snuff to 7,218,242*l.* 'Now,' says Mr. C. Morrison, 'without going the same length as those who would proscribe stimulants altogether, it may be assumed without much contradiction that nine-tenths of the spirits stated in this estimate, one half the beer and porter, and half the tobacco and snuff, were either actually pernicious in the use, or, at least, superfluous.' (p. 43.) The evidences of the amount of crime and pauperism fluctuate so much with the state of the law and its administration, that probably no standard of the moral and intellectual condition of our population is so sure as this inordinate and unnecessary use of intoxicating drinks and tobacco. This is fatal proof that, though the pauper bound by the law of settlement is our last actual slave, some of the worst consequences of serfdom remain. The workman is free to emigrate from the moorland cottage, or the hut in the fens, or the mud hovel in a stagnating agricultural district, or from an Irish bog. He may settle in the midst of the vigorous life of the English coalfields. He may be trained gradually with better food, lodging, and clothing, by the discipline of toil, till he has a muscular, energetic frame for outdoor work, or has gained a wiry, highly nervous organization for the skilled manipulation of the factory. He may submit his will to the regulated system of the division of labour and to the influence of opinion on society. He may combine with perfect respect to the law to adjust the relations of labour and capital. But, though in all this there is gain, the animal nature of the ancient serf reappears. Ages ago his progenitor was a beast of burden, sold as a chattel of his owner, when life was subject even to his caprice. He has not risen to real freedom until he has acquired self-control. Emancipated from the *mund* of his lord, he has become his own master without the power to control his appetites. In his new life the mind of the workman is mainly developed by his industrial and social training. The workshop and the press have done more for him than any other agency, and, next to these, the Sunday School. But it is to be confessed that that portion of the workmen who spend their evenings in sensual excesses have not yet become freemen. The tendencies to democratic changes are so obvious, and are so strongly indicated by the origin, history, and theory of our constitution, that they are in some form ultimately irresistible. We have, therefore, to determine for ourselves whether we value the personal liberty of opinion and action which we enjoy under the mixed political power of this country, and whether we cannot preserve it, while we proceed to fulfil the apparent destiny of our race, by completing the freedom of the mass of our countrymen, by raising them to the dignity of freemen in the power of self-control, and to the intelligent exercise of the rights of freemen by the recognition of their claim as a class to a more direct influence in our representative system. Something has been done towards this result. The cities, towns, and villages of our coalfields into which in the last century the population have migrated, were irregularly constructed, unpaved, unsewered, the houses often rude and unhealthy. There was bad scavenging, little lighting, no sufficient water supply. Though we had suffered from the warnings of typhus and of an excessive infantile mortality, we needed to be aroused by the visitation of cholera to the condition of our towns. That disease shocks by the appalling mystery which shrouds its advance, the rapidity of its action, and by the suddenness with which it ravages the population. The singular manner with which it marks by its path where the foulest squalor, the thickest miasm from filth attacks the frame wasted with want, and the deepest moral degradation, combined with the lowest physical condition, herd together, attracted public attention to the sanitary regulation of our towns. Great advance has been made during the last twenty years in these forms of improvement both in town and country. The physical condition of the people has also been greatly ameliorated by the cheapening of food and clothing and all the other necessaries of life, while their habitations and wages have improved. The protection of women and children under thirteen from excessive hours of labour, the prohibition of the employment of women in mines, have had a practical effect beyond the mere letter of the law. Excessive hours of work for men are discountenanced by public opinion; factories and mines are subject to regulations for the protection of life and health; and what the law does not require, an intelligent sense of Christian duty often effects. The new hamlets, villages, and towns have in the last half century, and especially in the last twenty-five years, been organized as centres of Christian influence by the building of churches, and chapels, and schools. We have spent many millions on these buildings. We probably now expend about 2,000,000*l.* annually on the education of the people. In the last quarter of a century literature has been cheapened for the use of the masses by such societies as that for diffusing useful knowledge, over which Lord Brougham has presided since its origin. The press has become the great instructor of the people in all social and political topics. An earnest practical effort has been within the same period made to foster in the working population habits of prudence by savings-banks, in which 30,000,000*l.* are accumulated; by building and benefit societies, in which large funds have been accumulated; by the possession of cottages and small freeholds; by temperance leagues; by societies of mutual improvement and mechanics' institutions; by advice, remonstrance, and example. The chief object of this brief review of the social history of the most numerous class of our fellow-countrymen will have been attained if it tend to inspire a lively faith in their destiny; if it teach us to recognise in our history how all the elements of our social state inevitably react on each other; how each advance in order, in peace, in social polity, in civil and religious freedom, in the power of mind over matter, and especially in the divine influence of Christian charity, has slowly but surely emancipated our humblest classes from serfdom, from villenage, from pauperism, and now tends to lift them up to the enjoyment of all the privileges of intelligent freemen. Nor have I been without hope that while such a review may thus strengthen our faith in the beneficent tendency of all providential laws, we may in contemplating their operation learn to restrain a short-sighted impatience. 1500 years have elapsed in our history, and yet the theory of our Saxon constitution is only partially realized. The schools of Edward VI. and Elizabeth only partially educate our middle class. Some generations must pass before home education in the cottage will generally worthily co-operate with the elementary school. How long a time will be required before the vast annual waste on intemperance is converted either into a means of rational enjoyment, domestic comfort, or into capital for the elevation of those who work with their hands! Whatever be the time required, we have to maintain our faith in the beneficent tendency of all great providential laws, whether in the great eras of material forces and animal life, or in the epochs of social change. All history teaches us that as the earth was in the vast ages of geological development slowly prepared by one great design for the habitation of man, so in the history of our race whatever have been the catastrophes which have overwhelmed empires—the internal ferments which have appeared for a time to cause a social chaos—slowly but surely, in the eye of Him with whom a thousand years are but as one day, man has been making a conquest of nature, asserting and exercising the dominion of mind over matter, emancipating himself from debasing animal instincts, raising class after class from serfdom, ignorance, and brutishness, and preparing for that reign of Christ in the hearts and institutions of mankind, when every man shall sit under his own vine and his own figtree, none daring to make him afraid."

The comments of the *Times* (October 19th) upon Sir J. K. Shuttleworth's paper form too instructive a pendant to be omitted in our *Retrospect*.

"It must be remarked that history has the infallible result of making a nation self-contemplative. The kind of retrospect which we have in this paper is the act of a nation thinking of itself, reviewing its own career, estimating its own success, and drawing its own character. What is the difference, then, between this self-estimating stage in Great Britain in 1859 and the same stage in the Athens of Pericles or the Rome of Augustus? There comes in the history of every nation, after a long course of *action*, in which it has done wonderful feats of all kinds, a *reflective* epoch, when it begins to be conscious that it has done all these great things, and to repose with self-congratulation on the sense of its own greatness. When Nebuchadnezzar looked down from the loftiest walls of the habitable world, and said, "Is not this great Babylon that I have built?' that was a national reflective act, for the monarch probably represented the pride of a nation, as Louis XIV. did. So Rome in the age of Virgil and Horace is always talking about herself, always indulging in historical vistas and the sense of her own majesty, always prophesying her own eternity. But this self-contemplative epoch in nations has generally been the sign of ebbing action—the fatal mark of corruption, effeminacy, and weakness having set in, of a nation being quite satisfied with what it *has* done, thinking it has done enough, and resting on its oars till some strong race that has yet to win its way pounces down upon it. What is the difference in our own case if, as we trust we may say, this is not the result with us—if the self-contemplative stage with us is not an effeminate one, but as active as any stage before it—if the self-congratulating look on the past is no omen of a feeble future?

"The difference is, that the civilization of these nations of the Old World was a very imperfect civilization, and, in fact, very little better than a mask for barbarism. It was founded on one single narrow virtue, and that is military virtue. The civilization that rose upon such a basis simply meant that a nation, having robbed every one about it right and left, and heaped up a huge mass of splendour and magnificence as the fruit of its spoil, began to indulge in gold vessels and marble baths, and to form some new experiences and tastes of a more delicate kind, such as the possession of wealth and leisure leads to. Such a civilization as this in its very formation totters upon a precipice, and becomes in the very next age what the historian calls 'luxury,'—that is, a corruption of the national spirit. When conquest is over, no other stimulus to exertion is left, and the monster, gorged with prey and soothed by repose, is ready for the dart. But the great characteristic of modern, at any rate of English civilization—and it is one which Sir J. Shuttleworth's paper exhibits strikingly —is the variety in its basis—that it has so many strings to its bow—conquest, freedom, labour—which have all contributed to produce our national progress. Conquest has united and concentrated us; freedom has organized us; labour has given us our wealth, and our leisure, so necessary to intellectual advancement. But the cardinal distinction lies in the last of these three—that our civilization has been a civilization of *labour*, and that is the great moral of Sir J. Shuttleworth's paper. He describes a triumphal march of labour, taking advantage of every opening, and never allowing itself to be absorbed in military show. Here is a natural principle, then, for civilization to rise upon, an ordinance of Providence, and not a mere stimulus of vanity, like the pursuit of conquest for its own sake. And the advantage of such a principle is that it supplies a *permanent* motive to human exertion. The love of work never loses its strength and charm as a motive: it has no natural limit, as conquest has; it goes on after it has built up the most enormous national capital, to found fresh and fresh capital; it is an indomitable motive, and it is a motive which always has a field for its gratification.

That is the reason, then, why our modern civilization does not verge upon national decay, as ancient civilization always did, and that we Englishmen in this year 1859 can indulge the self-reflective act, not as an idle triumph and effeminate repose in the past, but as the pledge of a future of action. Our civilization is one of natural labour, and there is no reason why we should ever leave off natural work. The field we have chosen is inexhaustible; it can occupy industrial Alexanders and Cæsars *ad infinitum.* There is not the slightest fear that we shall have more suns than we can do with, and that when conquest wants to go on worlds will stop. We have indeed some depressing mementos even in the midst of these cheerful and active prospects; the prophet's eye sees some clouds in the horizon, and some melancholy notes escape from his instrument while he is in the very process of encouraging and congratulating us. It would seem such a civilization as ours has its difficulties, and that the principle of labour, judging from the actual facts attending the trial of it, has not shown itself hitherto adequate to the task of producing a perfect civilization of the whole mass. It puts a great obstruction in the way of education, absorbing the youth of the country, and carrying them off from the school before they have fully mastered even the rudiments of knowledge. Sir J. Shuttleworth evidently looks forward to a long struggle of education with labour when he says "Some generations must pass away before home education in the cottage will worthily co-operate with the elementary school." But labour has a worse defect even than this. It does not tame the coarse sensuality of man, but leaves him too often a barbarian in the sense of living a prey to gross vices. "The operative," says Sir James, "may submit his will to the regulated system of division of labour; he may combine with perfect respect to the law to adjust the relations of labour and capital; but though in all this there is gain, the animal nature of the ancient serf reappears." Drinking is the curse of the working man. We may reasonably hope, however, that as the idea of education and of its necessity spreads in the lower classes, which it is now gradually doing, both education itself will advance and a powerful moral auxiliary will be gained against low sensuality; and that thus two additional triumphs, which are unhappily at present wanting to it, will be gained for English civilization."

Notwithstanding this hopeful, and we think truthful view of future triumphs of English civilization, the *Times* itself, bewildered with the complex details with which it is requisite to pave the way to, or to secure the prospective successes, has not altogether avoided the error which we commented on at the beginning of the retrospect. The mist of the present will at times becloud the light of history. But a few days after the article we have just quoted, the *Times* (October 27) wrote thus, respecting the charge of the Recorder of Birmingham.

"The charge of Mr. M. D. Hill to the Grand Jury at Birmingham will be found in another portion of our columns this day. There are many excellent and highly competent persons in this country who devote their attention to the culture of criminals, exactly as Dutch horticulturists give themselves up to the cultivation of tulips. The tulip fanciers as yet have had the best of it, although they have not succeeded in producing that black specimen which is the chief object of their desires. Still they have had the best of it, although, from the days of the illustrious Howard to our own, England can show a bright list of philanthropists who have devoted every energy of their lives and every faculty of their minds to this sacred cause. The good they have effected is great; the mischief not inconsiderable, perhaps, but unavoidable. When we burst loose from the old theory, so dear to the heart of the late Lord Ellenborough, that the only method of dealing with crimes was to hang, banish, flog, or imprison the criminal, our course was a very simple one. The old cry was "More crime, more hemp—more hemp, more crime!" That was the good old system of the hanging Lords, who were never in want of the fag end of a verse from *Leviticus* to justify their wholesale strangulations. The whole system was so dreadful, so entirely calculated to defeat its own ends, that men of this generation can only wonder that no one rose up before Romilly to protest against it. It became evident at last that we were dealing with the symptom,—not with the disease itself. The more we strangled our fellow-countrymen, the more they would filch pocket-handkerchiefs; it was very astonishing, but so it was. What wonder, after the wholesale executions which disgraced the practice of our fathers, if the wheel in our hands has flown too much round in the opposite direction? Where they tortured and slaughtered persons who had been guilty of some very trifling offence, we have taken atrocious criminals, and dealt with them as though they were little lambkins who had gone astray; we have endeavoured to work upon their sensibilities and pampered them in comfortable lodgings which had nothing about them of a prison but the name. So great and so deplorable was the reaction against the course pursued by the judges of the Ellenborough school. It would, however, be too much to say that the criminal population have been entirely the gainers, considering the commission of crime only as a matter calculated to promote their own immediate comfort. When it became clear that neither Draco nor Jean Marie Farina was the right person to deal with our criminals, we handed them over to one experimentalist after another, who engaged to wash our Ethiops white, and to conjure the spots from the backs of our social leopards. In many of these attempts—well and humanely intended as they, no doubt, were—they have inflicted greater misery upon their patients than the old practitioners, who knew but one remedy—the sudden infliction of physical pain, and the deprivation of life. We have tried silent systems, solitary systems, systems with instruction, systems with premiums for good conduct, and systems where cant was at a premium. We have endeavoured to keep the old and the young apart, to separate the novice from the hardened offender. Except in very extreme cases, we now retain our criminals at home, and make them the subject of one experiment after another. For all this the principles of the science are as yet scarcely established.

Among the gentlemen who have honourably distinguished themselves in these humane and praiseworthy attempts to deal scientifically with our great social plague, Mr. M. D. Hill, the Recorder of Birmingham, occupies a very prominent place. We know of no one who has shown greater patience and perseverance in this cause, and who has more entirely devoted his life to it, than Mr. Hill. Now, let any one read his charge carefully—we print it to-day —and he will find that after all the experiments that have been tried, all the efforts that have been made, and with all the advantages of long experience to guide him, Mr. Hill arrives at the conclusion which was already familiar to simpler men. Just as the preacher at the end of his career wrote "Vanity, vanity, all is vanity," so Mr. Hill tells us, as the result of his mature reflections upon this subject, "Drunkenness, drunkenness, all is drunkenness." Keep the poorer classes from the abuse of intoxicating liquors, and you will cheat the hangman of half his work, and be enabled to diminish your gaols by about eighty per cent. When Model Prisons and Reformatories and what not have done all that could be expected from them, and every philanthropist has tried his favourite panacea, we have this great black fact staring us in the face—that drunkenness is still the great "*causa causans*" of English crime. Mr. Hill besides this, adverted to another point well worthy of the attention of all persons who are investigating this painful subject. It is undoubtedly a truth that in certain respects crime and destitution go hand in hand. In hard times with low wages and scanty employment, certain crimes proportionately increase. The law appears to be as fixed as any natural one. There is a large portion of our population just oscillating between destitution and a bare sufficiency. When they have this bare sufficiency they remain honest; when they are stricken with destitution they pilfer and thieve. So it was twenty years ago, so it was ten years ago, so it was one year ago, and so it is now. While we are discussing the subject of crime and the treatment of criminals we must, then, never lose sight of these two cardinal points—that he is the best criminal reformer who can do aught to promote sobriety among the humbler classes, and in any way help forward the general prosperity of the country. The gentlemen who assisted in the repeal of the restrictive laws upon food were great criminal reformers—the persons who have led our poor misguided artisans into this wretched system of Strikes frightfully the reverse.

We would, then, impress these two points upon the attention of our criminal reformers as the alpha and the omega of the science they have taken in hand. Deal with these, and you deal with the roots of the evil. Other measures may be very useful and very necesssary, but they are merely palliatives. We are glad to hear from Mr. Hill's lips that reformatories have effected all the good that could be expected from them, and we hope that it is so. He tells us, also, that the magistrates in the manufacturing districts have found out by practical experience that the system of short sentences is more calculated to promote than to check crime. The inference is, that long sentences are found to check the commission of crime, and so far the result is satisfactory enough. When we add to this that Mr. Hill's practical experience has shown him that there must be a liberal scale for payment to witnesses and others engaged in criminal prosecutions, we have pretty well exhausted the leading points of his charge. He deals, indeed, with a few minor matters, and gives some recommendations which may be considered the stereotyped formulæ in use among criminal judges when the occasion arises for their use. Mr. Hill, however, in the course of his charge mentions that in his district—Birmingham —and, indeed, throughout the country, there is a diminution of fifteen per cent. in the convictions. All necessary allowances being made for confusion between the number of convictions obtained and of crimes committed, Mr. Hill has satisfied himself that there is an actual diminution of crime in his district. This he thinks due to improved methods of dealing with the criminal population, and not merely to transitory causes. It would seem at last, after all the noise which has been made about the treatment of our criminals, that we have arrived at a sound principle or two. Such a result was much wanted, for there was a growing spirit of disbelief in the value of exertions which had been so long continued without any apparent effect.

Has our experience in these things been so long that it justifies a serious doubt? Has not our anxiety to witness the results we hope for rather led our anticipations beyond legitimate bounds? Have we not too often confounded the reformation of criminals with the reformation of the sources from which they come? We may congratulate ourselves, we think, that our efforts are beginning to tell effectively upon the actual criminal, and to restrain the criminal population within certain bounds. This we learn from our judicial statistics, and what more could be anticipated from our prisons and police, which act only upon overt crime and not, except indirectly, upon the substratum from which it springs. We now see our way more clearly to act directly upon this. We learn from every trustworthy source that intemperance is one of the most important fertilizing causes of crime. Here there is one spring to be stopped up, but we have already been told by Sir J. K. Shuttleworth that this will probably be a labour of generations, not of the moment. We must learn, as he has admirably said, to restrain a short-sighted impatience. It will be long before we can control intemperance, but "whatever be the time required, we have to maintain our faith in the beneficial tendency of all great providential laws, whether in the great eras of material forces and animal life, or in the epochs of social change."

It is an easier task to note the changes which occur, and the circumstances which induce them, in isolated segments of, than in an entire nation. The questions presented are less complex but not less important, because their solution affords those fundamental data without which our attempts to solve the vaster problems of social changes would be futile. We have now lying before us a recent work* on the asserted decadence of Quakerism which is of considerable interest from this point of view. The work is of a controversial rather than of a scientific character, but the sources to which the decay of the society is attributed by the author are of considerable interest to the psychologist. These are as follows:—

"1. The visionary, inappreciable, and yet dangerous, character of the fundamental doctrine of the society, which gives precedence to the 'inward testimony of the Spirit,' and not to the 'revealed scriptures.'

"2. The rejection of an organized and paid ministry; and the recognition of the immediate inspiration of male and female preachers, for the various occasions of devotional assembling.

[1] "A Fallen Faith, being a Historical, Religious, and Socio-Political Sketch of the Society of Friends." By Edgar Sheppard, M.D. London. 1859.

"3. The unsatisfying and unsubstantial character of the society's form of worship.

"4. An absolute physical, mental, and moral deterioration, arising from the combined effects of frequent intermarriages, morbid 'seriousness,' and a too emotional and introverting religion.

"5. The singularly obstructive character of the society's socio-political relationship to the world—comprising the questions of the lawfulness of oaths, of war, and of ecclesiastical imposts; the peculiarities of dress, language, and deportment; the prohibition of sports and amusements; the non-recognition of distinctive social inequalities, and the prohibitions placed upon trade.

"6. A general recognition on the part of the best Friends of the necessity of a higher educational standard, adapted to the requirements and the progressive spirit of the age; this recognition involving, as a matter of course, a repudiation of their 'ancient principles,' a fraternization with the world, and a consequent separation from themselves.

"7. The exclusive character of the society, and the system of disowning members for breach of discipline; and its non-proselytism.

"8. A growing conviction on the part of the most educated and enlightened Quakers, that the notorious material prosperity of the society does not accord with its exalted spiritual profession, though it does not absolutely militate against the moral character of individual members."

Dr. Sheppard lays considerable stress upon the mental deterioration of the Quakers and the insanity resulting from it; and although his remarks are chiefly based on *à priori* reasoning, they are very suggestive. Whether or not they may prompt the society, which holds so honourable a position in the history of the non-restraint system of treating lunatics, to test their accuracy or not, we cannot say. We know too well from harsh experience how difficult a task it is to determine any body of individuals, as well as the public at large, to look at these matters with common practical good sense. It usually requires something of a cataclysmal nature to provoke a large society of individuals or a people to self-reflection upon subjects which are not immediately within the reach of all apprehensions. We would fain hope that certain recent grave events will have the good effect of rousing the press and the public to more sober reasoning and conclusions respecting insanity at least.

In our last *Retrospect* we recorded the horrible murder of a young girl by a gentleman named Pownall, who had been a few days before the event discharged from a private asylum. In our present retrospect we have to record another murder which was perpetrated under somewhat similar circumstances. On the 28th November last, an operative named James Moore, murdered his wife (in Finsbury), and defaced the body in a most frightful manner. On his trial it was deposed that he had been discharged from a lunatic asylum about a fortnight or three weeks before the deed. The following medical evidence was given during the trial.

Mr. Dixon, the medical attendant of Hoxton Lunatic Asylum, deposed that the prisoner was under his care from August, 1858, until November, 1859, when he was discharged as cured, and, in his opinion, at that time the prisoner was perfectly sane. The prisoner, when he first saw him, was labouring under mania and had several delusions, one of which was that he had a steam-engine in his inside. For a considerable time he was prone to violence, quarrelsome, and easily excited. He began to get better in September, and was very anxious to be discharged, in order that he might work for the support of his wife and family. The deceased used to visit the prisoner once or twice a week while he was confined in the asylum, and he appeared very kind and affectionate to her, and seemed very anxious to be discharged, in order that he might work for her support.

Mr. Gibson, the surgeon of Newgate, was then examined, and he stated that the prisoner had been under his observation for more than a fortnight, and the opinion he had formed of him was that he was of unsound mind when he was brought into the prison, and had remained so ever since. He had some conversation with the prisoner relating to his crime, and in the course of it he asked him if he did not regret what had occurred. He replied that he did not, and he said he believed it was only part of a plan to get him back into the lunatic asylum.

In answer to a question put by Mr. Sleigh,

Mr. Gibson said that from what he had seen of the prisoner and the other facts in the case, he had formed the conclusion that when the prisoner committed this act he was not of sound mind.

The conclusion of the case is thus stated:—

The prisoner, having insisted upon addressing the jury, made a long rambling and incoherent statement, the principal object of which appeared to be to show that he was of perfectly sound mind. He said that when he was originally sentenced to nine months' imprisonment he was healthy and strong, and able to bear the punishment; and nobody had a right to send him to a lunatic asylum. As to the crime of which he was accused, he said that no one saw him do it, and with regard to the evidence as to the blood on his clothes, they were second-hand clothes, and the blood might have been on them when they were purchased.

Mr. Baron Watson having summed up,

The jury retired for a few minutes to deliberate upon their verdict, and on their return into court they gave a verdict of *Not Guilty*, on the ground of insanity.

The learned Judge ordered the prisoner to be detained during Her Majesty's pleasure. He was evidently very much displeased at the result, and seemed anxious again to address the jury, when he was removed from the bar.

The early history of Mr. Pownall's case, as made known during his trial, and related by the *Times*, was as follows:—

The prisoner at the time of the unfortunate occurrence was residing in the house of Mr. Leete, a surgeon at Lydney. He had only been recently liberated from the lunatic asylum at Northwoods as "cured," and, as our readers may recollect, he got up early one morning and cut the throat of one of the maid servants with a razor, and so caused her death within two or three minutes. As the main question now was as to the state of the prisoner's mind at the time he did the act, we give only a brief narrative of the facts as proved by the evidence, beginning with the early history of the prisoner, as detailed by the witnesses called for the defence. It was stated that the prisoner had been several times in lunatic asylums, but it was thought necessary to trace his history only from the month of March last. The prisoner was then residing at Wroughton, near Swindon, with his wife and sister, and wife's mother, an old lady between eighty and ninety years of age. In that month he was seized with what the doctors called homicidal and suicidal mania. He attacked his aged mother-in-law, and beat her about the head with a poker, till he had nearly killed her, and then attempted to destroy himself. Dr. Morris, a physician at Swindon, was called in, and found the prisoner, Dr. Pownall, sitting in a chair in the kitchen in a state of insensibility, and suffering from the effects of some narcotic poison, which, from a bottle which was shown to him, the witness believed to be chloroform. External and internal stimulants were used, and the prisoner became sensible. The same day Dr. Morris saw him again, when the prisoner said he was better, and that, unfortunately, he had taken chloroform, but he was sorry for what he had done, and wished it not to be mentioned. He said he had been annoyed by Mrs. Pownall and had met with pecuniary losses, and his object was to destroy himself. He also said they excited and upset him, and he hit the old woman on the head. From what the witness saw and was told by Mrs. Pownall, he signed a certificate for his admission into Dr. Davey's asylum at Northwoods. This witness, in answer to questions from the learned judge, explained that in homicidal and suicidal mania the impulses are sudden, and are often followed by tranquillity as soon as the impulse has been gratified. The other medical man who signed the certificate was a Mr. Gay, but he was not called as a witness. Charles Bonham said, he was called in and saw the cuts on the head of the old lady, and arranged to sit up at night with the prisoner. One night he was sitting up with him, with Dr. Davey's son and a keeper, and it appeared the prisoner overheard some conversation which had taken place, and observed that he saw through the trick. He then went into a long statement, complaining of the conduct of his family to him, and continually complained of his food being drugged. He became much excited when he knew that Dr. Davey's son and the keeper had come, and managed to get out of the room, under pretence of calling for some water, and returned with a double-barrelled gun behind his back, and rushed with it to the room where his wife, sister, and mother-in-law were, and pointed the gun at them. His sister seized the gun by the muzzle and lifted it up, and the keepers laying hold of him behind, the gun, which was loaded, was taken from him without any injury being done. The next day the prisoner was taken away to Dr. Davey's asylum, at Northwoods. Dr. Davey was examined, and said he received the prisoner with a certificate, which stated that he was labouring under homicidal mania, and he treated him accordingly. In about three weeks he appeared considerably improved, so much so that on the 17th of May Dr. Davey wrote a letter, in which he said he thought he was in that state of mind in which he would be improved if he were restored to his liberty, but he (Dr. Davey) found an unwillingness on the part of the prisoner's family to his being restored to liberty. Some correspondence ensued with her Majesty's Lunacy Commissioners, who wrote a letter to Dr. Davey, which he had destroyed, but in which, he said, the Commissioners required that the prisoner should be restored only, under leave of absence, under a keeper. The family wished the prisoner to be removed to the asylum at Coton Hill, near Stafford, but Dr. Davey said he could not give a certificate of insanity for him to go there. The prisoner was detained at Northwoods till a suitable attendant could be engaged to go with him; but this difficulty being at length got over, the prisoner was taken by his new keeper, named Richard Pook, to the house of Mr. Leete, at Lydney. When Dr. Davey discharged the prisoner, he gave him a certificate in the following form :—

" 'Northwoods, near Bristol, 10th of August, 1859.

" 'I hereby certify that Dr. Pownall has been under my care here for some four months, that he is now quite recovered, and is this day discharged cured.

" 'J. G. Davey, M.D.'

"Dr. Davey said he had no doubt that the prisoner, when in his asylum, had been suffering from homicidal mania, and that when he committed the act now in question he was suffering from a paroxysm of that disease, and was unable at the time to distinguish right from wrong. He said he gave the certificate above referred to to the prisoner, in order to influence his mind hopefully and cheerfully, and so to put him in the best possible state for recovery. He never allowed the prisoner to have razors, and he did not think it necessary to give Mr. Leete any caution on that point, as he supposed the prisoner was going to be placed under the care of a gentleman conversant with cases of insanity, who would not allow his patient to have razors. Dr. Davey also said that at the same time that he gave the prisoner the certificate that he was 'quite recovered' and 'cured,' he also gave him a letter of introduction to Mr. Leete, containing certain hints 'to put Mr. Leete on his guard.' Instead, however, of sending this letter by the post, Dr. Davey gave it to the prisoner, who produced the certificate to Mr. Leete, but said nothing about the letter of introduction, and nothing more was seen of it till it was found among the prisoner's papers in his bedroom, after the dreadful tragedy had been enacted. The prisoner arrived at Mr. Leete's, at Lydney, on the 10th of August, and soon gave Mr. Leete to understand that as he had got a certificate of cure he was a free agent, and would not be under control, and, on the 23rd of August, he dismissed his keeper, telling Mr. Leete that he did not want him (the keeper), and that he himself was going to London in a few days to settle some business. The prisoner had always borrowed a razor of his keeper, who used to lock it up immediately it was done with, but after the prisoner had discharged his keeper, his razors were given to him. The prisoner's friends, when applied to on the subject, objected to his having them; but on the 8th of August Mr. Leete wrote them a letter, in which he said, 'I consider all the precautions requested to be taken with respect to Mr. Pownall to be perfectly unnecessary.' The razors were accordingly given him, and, as he had money, the prisoner was allowed to go in and out, and to conduct himself entirely as if he were a free man and in his perfect senses."

The subsequent history of the case we gave in our last *Retrospect*. Mr. Pownall was acquitted on the ground of insanity.

Now we confess that we are not much surprised at the occurrence of these murders. The tone of public opinion in reference to the detention of lunatics in private asylums, and the mode in which that opinion has been manifested once or twice in courts of law during the last three or four years, has been well calculated to bring about such results as those witnessed in the cases of James Moore and Mr. Pownall. It was hardly to be anticipated that the anathemas which have been again and again launched by the press of late, against the supposed unjust detention or unrighteous admission of individuals into private asylums; the persistent vilification of medical men practising in insanity and of the proprietors of asylums; and the manifest influence which this popular clamour exercised upon the pleadings at the bar in cases where the question of lunacy was raised, upon juries, and even upon the bench, would not have a greater or less influence upon the conduct of medical men and of the proprietors of asylums. It might have been expected, as a natural result of the tendency of public feeling, that the proprietors of asylums would become very chary of detaining a lunatic one moment longer under care than was absolutely necessary; and that a medical man would not be in haste to give a certificate of insanity unless there were symptoms present which might satisfy a jury. This latter course of action became, indeed, almost a necessity after the Right Hon. the Earl of Shaftesbury, the Chairman of the Lunacy Board, had authoritatively stated before a Parliamentary Committee, a few months ago, that the opinion of a man of ordinary observation was better than that of a medical man in determining the question of insanity. Under these circumstances, if a professional man would keep clear of a sharp lawyer and the possibility of being sued for damages for illegal detention of an individual, or for signing a certificate unjustifiably, it was necessary that he should adapt any public manifestation of his knowledge of insanity to the level of information possessed by the bulk of the people, and that he should detain no case under care in which symptoms were not present that could not be readily made apparent to a jury, and which did not approximate to the popular notions of insanity. This has in reality come to pass. Medical men and the proprietors of asylums have been obliged to give way for the moment to the popular clamour; and as a result we know that, for the necessary security of the proprietor, it has been requisite to dismiss from asylums and from superintendence, cases of insanity accompanied with dangerous and destructive tendencies, but which cases have been characterized by long fits of intermission. James Moore's case constitutes the third instance of murder committed by individuals recently discharged from asylums, within a period of eighteen months in this kingdom. In the first of these cases the dismissal of the lunatic arose solely from the necessity occasioned by the popular feeling. The man was dismissed, and although he had been several months in the asylum previous to dismission without manifesting any sign of insanity (his detention resting solely upon the fact of the known dangerous nature of his delirious paroxysms when they occurred), within fourteen days after dismissal he had stabbed a man mortally in the public street. We cannot help regarding the dismissal of Moore and Pownall from the asylums where they had been under care, as having been brought about in no small degree by the influence of the popular outcry to which we have referred. But, alas! murder is one only, and the rarest, of the evils induced by a too early dismissal of a lunatic from charge. Who can tell the domestic misery and ruin which have been too often occasioned by the revengeful or extravagant lunatic, who, *quasi*-rational and harmless within the walls of an asylum, has at once manifested his insane fancies and feelings when restored again to his family and to self-control? The indiscriminate reprobation which has of late been heaped upon private asylums, so far from tending to remove such evils as may still belong to them, has, we believe, given rise to several additional and even graver evils.

Accustomed as we have been to the animadversions of the public, and the sneers of the bench upon the so-called crotchets of medical men, who, from time to time, have come forward in order to rescue a lunatic from the scaffold, it is refreshing to find a judge publicly admitting that an attempt to save a man from being hanged on the ground of insanity may really arise from a most worthy motive. The instance perhaps smacks somewhat of the principle—

> "That in the captain's but a choleric word,
> Which in the soldier is flat blasphemy."

A short time ago a Portuguese sailor named Charles Annois committed a diabolical and most inexplicable murder in a British ship on the high seas. He was tried at the Central Criminal Court, and sentenced to death. The motiveless character of the deed, and almost entire ignorance of the man's history previous to his joining the ship at Lisbon, which was but a very short time before the murder, led, however, to the suspicion that he might be a lunatic. Apart from the circumstances attending the deed, no traces of insanity could be detected, but there was some reason to believe that the man had been confined as a lunatic at Lisbon. *After* the trial and sentence, the prosecuting advocate, Mr. Sleigh, waited upon Mr. Justice Williams, and submitted to him certain doubts, "and asked him if he considered it would be acting in any way unbecoming his position, if he were to wait upon the Home Secretary, and have some communication with him in reference to the fate of the unhappy prisoner. Mr. Justice Williams stated that it appeared to him that, so far from the interference of the learned counsel being in any way unbecoming or improper, he considered that the interest he took in the fate of the unfortunate prisoner was highly creditable to his humanity, and honourable to him in his position as an advocate; and that *if he felt that the prisoner's life should be spared, it was his duty* to put the Secretary of State in possession of every information that would justify him in coming to that conclusion." When shall we hear a judge address such language to the medical man who, as a matter of duty, comes forward to testify to such things as, in his opinion, prove the criminal irresponsibility of a person who may have been guilty of an illegal deed? Mr. Sleigh had an interview with the Secretary of State, and the murderer is now under respite until some account of his life previous to his being shipped on board the British barque can be obtained.

The legal progress of this case, and the popular estimate of it have been altogether curious. The *Times* (Oct. 28) had the following comments upon it:—

"A Portuguese seaman was convicted the day before yesterday at the Central Criminal Court of the wilful murder of his captain. The murder occurred on board a British ship called the *Margaret;* she was eleven days out, on her way from Lisbon to a port in North America, when the crime was committed. The facts of the case are very simple, and we will presently give a brief recapitulation of them. The only noteworthy point in the trial, beyond the interest necessarily inseparable from the details of so atrocious an act, turned upon the question of the sanity or insanity of the prisoner at the time. The act was the act of an insane person, but for all that appeared in evidence the prisoner's antecedents were in no wise tainted with insanity. Argue from the prisoner's life until the moment when he committed the crime, and he was sane enough; argue from the act itself, and he was as clearly insane as any lunatic prisoner now confined in Bedlam. The history of his previous life was certainly not known in a satisfactory manner, for he appears only to have come on board the ship on the 28th of July last, and on the 21st of August he committed the crime for which he was condemned. Under what circumstances his previous life had been passed, whether or not tokens of insanity had cropped out during his youth and early manhood—he is but twenty-five years of age,—there was nothing to show. The jury under these circumstances took the common-sense view of the case, overruled the plea of insanity, and found him guilty of murder. It would, of course, have been more satisfactory if we could have obtained some knowledge of the details of his previous life; but the criminal is a native of Rio Janeiro, and we presume there were insuperable difficulties in the way, or this line would have been taken for the defence. We cannot but regard the finding of the jury with satisfaction; for there has been of late years far too much of maudlin sympathy with heinous offenders, far too great a tendency in jurymen to shirk the responsibilities of their position, far too great a readiness to compromise between their feelings and their judgment, by bringing in a verdict which, at the same time that it spares them the necessity of consigning a human being to the scaffold, will, as they suppose, preserve society from any repetition of offences committed, at least, by that one hand. . . . . . .

"——Of course, the only doubt could be as to the condition of Annois' mind at the time the murder was committed. The ancient rule of English law is, that a man is not criminally answerable for any act he commits if he be not capable of distinguishing between right and wrong. More than this, any delusion entertained by a prisoner must be proved to influence the act, or it is not material. The defence appears to have been that the crime was of so atrocious a character that the prisoner must have been mad when he committed it. This, however, would be a most dangerous precedent, for the bare atrocity of an offence would then carry impunity with it. Now, there is not one word of evidence to show that the prisoner was not aware of the difference between right and wrong, or innocence and guilt. When questioned, the day after the crime, what motive had induced him to commit it, he stated it as his reason that the captain had given him too much work. All the witnesses most distinctly swore that they had not observed in him any indication of insanity previous to the murder of Captain Barker. Equally important perhaps is it to remark that the prisoner's counsel did not pretend that he was of insane mind at the time of his trial, or that he had given proof of insanity in the interval between the murder and the trial. If the man was mad, he was mad on the night of the 21st of August last—neither before nor since. The jury, happily, would not listen to such a plea; their sympathies were of a more wholesome kind than those which too commonly have influence with the jury-box. Their pity was not for the murderer, but for the innocent victim of his revenge."

The question of sympathy with the murderer, or the murdered, is a matter of feeling which need not trouble us here, as we have simply to deal with the matter of fact. Was the murderer a lunatic or not? It is an axiom in law and in physic, that the act of murder or suicide, *per se*, cannot be received as a proof of mental unsoundness. But it is not the less true that the character of the act—the mode of its perpetration—may lead one to suspect insanity. This was manifestly the case in Annois' case, as shown by Mr. Sleigh's subsequent conduct. When the suspicion arises, it would appear to be plain that we should inquire carefully into the antecedents of the murderer, in order that the doubt should be solved. This seems to be a simple common-sense proceeding enough, but who can fathom the profundities of the law? Annois was brought to trial, tried, and condemned to death; advocate, judge, and jury being alike totally ignorant of the history of the man previous to the murder! This is startling enough, but what follows would be supreme in its whimsical eccentricity if the subject were not so serious. The leading journal sings an Io Pæan over the case, as a crucial instance of justice; and as a climax the advocate who had successfully prosecuted the case, and secured the conviction and sentence of the murderer, immediately afterwards seeks a respite for him lest he should be a lunatic! Would not the law have had a more decent aspect, and have borne a greater similitude to justice, if the trial had been postponed, and information necessary to determine the question of lunacy obtained first?

